# Assessment of Sleep Quality in Adolescents with Overweight and Obesity Using the Adolescent Sleep Hygiene Scale (ASHS)

**DOI:** 10.3390/children11111372

**Published:** 2024-11-12

**Authors:** Eleftheria Kampani, Eleni P. Kotanidou, Vasiliki Rengina Tsinopoulou, Evdoxia Sapountzi, Stergianna Ntouma, Evangelos Pavlou, Assimina Galli-Tsinopoulou

**Affiliations:** 1 Program of Postgraduate Studies “Adolescent Medicine and Adolescent Health Care”, School of Medicine, Faculty of Health Sciences, Aristotle University of Thessaloniki, 54636 Thessaloniki, Greece; ekampani@auth.gr (E.K.); epkotanidou@auth.gr (E.P.K.); vitsinop@auth.gr (V.R.T.); espavlou@auth.gr (E.P.); 2 2nd Department of Pediatrics, School of Medicine, Faculty of Health Sciences, Aristotle University of Thessaloniki, 54636 Thessaloniki, Greece; sevdoxia@auth.gr (E.S.); liandouma@yahoo.gr (S.N.)

**Keywords:** sleep, obesity, adolescent, ASHS, insulin resistance

## Abstract

Background: Adolescent overweight and obesity are a public health problem with an epidemic trend. There is growing evidence that sleep quality correlates to body weight. The aim of this study was to investigate, sleep quality in adolescents with obesity/overweight. Methods: A total of 100 adolescents with overweight/obesity aged 12–18 years were enrolled. Anthropometric parameters were recorded and a laboratory investigation in the fasting state [glucose, insulin, cholesterol (TC), high-density lipoprotein cholesterol (HDL), low-density lipoprotein cholesterol (LDL), triglycerides, uric acid and glycated hemoglobin (HbA1c)] was performed. Insulin resistance was calculated by the Homeostasis Model Assessment of Insulin Resistance index (HOMA-IR). Sleep quality was assessed with the Adolescent Sleep Hygiene Scale (ASHS) questionnaire. Results: According to ASHS, 93% of the participants were classified as “Good Sleepers” (GSs) (score > 3.8) and 7% as “Poor Sleepers” (PSs) (score < 3.8). PSs had a statistically higher body mass index (BMI) compared to GSs (*p* = 0.026). Increased body mass index (BMI) (r = −0.306, *p* = 0.002), fast insulin (r = −0.224, *p* = 0.027), and HOMA-IR (r = −0.260, *p* = 0.010) exerted a negative effect on sleep quality. Controlling for lipids and uric acid, only TC levels appeared to have a statistically significant and specifically positive correlation with the ASHS score (r = 0.202, *p* = 0.045). HbA1c values and waist circumference tended to be negatively correlated, but not significant to adolescent sleep quality [(r = −0.101, *p* = 0.330), (r = −0.095, *p* = 0.359), respectively]. The influence of central obesity on the ASHS score was also explored, but no correlation was found (*p* = 0.566). Conclusions: Sleep quality, as reflected by the ASHS score, was associated negatively with BMI, fasting insulin levels, and insulin resistance. Furthermore, a gender difference was observed, as adolescent males were found to achieve a higher overall ASHS score compared to females.

## 1. Introduction

According to the World Health Organization (WHO), the global prevalence of overweight among children and adolescents, aged 5–19 has increased dramatically from 8% in 1990 to 20% in 2022 [1]. The frequency of childhood overweight and obesity in Greece has been recorded at 40.4% for girls and 43.9% for boys [2]. Investing in adolescent health and well-being leads to a triple benefit over time: a benefit for the current adolescent population, for the next generation of the adult population, and for the generation of their offspring [3]. In the context of investing in the health care of adolescents, major efforts are focused on studying and documenting all the factors that threaten their health, among which overweight and obesity are prominent factors.

Comorbidities of obesity in adolescence include the development of a wide range of diseases such as type 2 diabetes mellitus (T2DM), dyslipidemia, hypertension, sleep apnea syndrome, nonalcoholic fatty liver disease, and others. The short-term effects of obesity on adolescent quality of life in terms of socialization, sports, and mental health are also strongly documented [4]. The most important long-term effect is the development of adult obesity, which entails an increase in both cardiovascular mortality and the incidence of malignancies [4]. Another factor driving obesity’s impact is the large financial cost. According to the World Obesity 2023 report, in 2020, the cost of obesity worldwide amounted to USD 1.96 trillion [2].

The etiology of obesity is broad and includes factors of both nature and nurture [5]. Nevertheless, the vast majority of cases of childhood obesity are of non-organic etiology, mainly related to the vicious lifestyle cycle of insufficient physical activity, consumption of processed foods with high calories, and sedentary life. Current data also correlate weight gain with hormonal disruptors [6], with the first 1000 days of the individual’s nutrition [7], and also with intrauterine exposure to maternal stress, undernutrition, or overnutrition [8]. Interactions between sleep and body weight are of particular scientific interest, as both reduced duration and poor sleep quality are associated with overweight and obesity [9,10].

Since the 1980s, evidence has been available regarding the role of sleep in human homeostasis [11]. Sleep is known to influence the immune response to both infection and vaccination, as well as the development and progression of cardiovascular disease and malignancy [12,13]. Sleep also participates in normal functioning of the brain, in the maintenance and replenishment of energy stores, in thermoregulation, in the formation of cognitive functions (e.g., consolidation of knowledge), and also in the behavior and psychology of people [11]. Inadequate sleep is nowadays recognized as a regulator of neuroendocrine functions and has been associated with increased cortisol levels in the afternoon, decreased glucose tolerance and insulin sensitivity, increased ghrelin levels, decreased leptin levels, and ultimately, increased appetite and overeating [9]. Circadian misalignment due to either insufficient sleep or poor sleep quality involves not only centrally secreted hormones such as melatonin but also peripherally secreted hormones, such as leptin, ghrelin, peptide YY, and glucagon-like peptide 1 (GLP-1) [14].

The association of sleep with glucose metabolism has been studied mainly through observational and cohort studies. The main mechanisms describing their relationship suggest that insufficient sleep acts as a stressor, activating the hypothalamic–pituitary–adrenal axis to release cortisol and promote insulin resistance [15]. Additionally, insufficient sleep also activates the sympathetic nervous system, leading to altered leptin concentrations and causing increased appetite and decreased satiety [6,7,8,9,10,11,12,13,14,15]. There is evidence that sleep disturbances participate in the altered methylation status of many Cytosine-p-Guanine (CpG) sites, therefore gaining a role in the epigenetic pathophysiology of several metabolic pathways multiplying the risk of obesity [16]. 

Sleep disorders, classified by the American Society of Sleep Medicine, are common and are estimated to affect 15–44% of children and adolescents [17]. Sleep-related problems during adolescence are mainly delayed sleep phase syndrome, poor sleep hygiene, insomnia, narcolepsy, restless legs syndrome, and obstructive sleep apnea [18]. In human physiology, the onset of puberty leads to a characteristic decrease in melatonin secretion, associated with a delay in sleep and wakefulness by approximately two hours [19]. Poor sleep hygiene appears to be an important component of sleep disorder, but there has long been no consensus on definitions. Elements of sleep hygiene include maintaining a bedtime routine, a stable sleep schedule, and avoiding screen use and caffeinated beverages in the afternoon/evening hours [20].

There is evidence on sleep and its impact on the mental and physical health of children and adolescents, focusing on sleep duration. Limited research specifically examines sleep quality in children due to children’s limited ability to be expressive at younger ages, in addition to the fact that sleep quality may matter more than sleep quantity [21]. Data examining the relationship between body weight and sleep quality and hygiene are very scarce. There is no evidence from validated clinical instrument questionnaires on sleep quality in children and adolescents with obesity. The aim of the present study was to investigate sleep quality in overweight and obese adolescents and to examine potential associations between sleep hygiene and central obesity, glucose metabolism, and lipid profile, rather than simply assessing sleep duration.

## 2. Materials and Methods 

### 2.1. The Study Population

The study population included adolescents who attended the Pediatric Endocrinology Outpatient Unit of the 2nd Department of Pediatrics of the School of Medicine of the Aristotle University of Thessaloniki, Greece, due to body weight excess. Inclusion criteria for this study were age ranging between 12 and 18 years and body mass index (BMI) ≥ 85th percentile according to the Center of Disease Control (CDC) growth charts for age and gender. This study focused on an otherwise healthy adolescent population with isolated obesity or overweight, without co-morbidities, in order to minimize the effect of covariates. Εxclusion criteria included conditions that could act as confounders in the relationship between sleep quality, body weight, and metabolic parameters.

A diagnosis of previous psychiatric disease was considered an exclusion criterion since sleep disturbances are related to the pathophysiology of the primary disease and thus represent a co-variable of obesity. By the same reasoning, adolescents with chronic diseases, genetic syndromes, active malignancy, history of malignancy, and substance abuse were also excluded. Patients with neurodevelopmental disorders were excluded due to their inability to participate in this study. Sleep apnea patients constitute a population where sleep is disturbed by the nature of the disease rather than sleep hygiene habits, so they were also excluded. Moreover, pregnant teenagers were not included in the present study as this is a period of major changes in both body weight and sleep habits. Patients with any acute infectious disease were also excluded, as the infection could affect the results of the laboratory tests performed. Finally, adolescents who received drugs affecting either sleep (β-blockers, non-steroidal anti-inflammatory drugs, diuretics, antidepressants) or body weight (corticosteroids) were considered ineligible for participation.

### 2.2. Anthropometric Data and Laboratory Investigation

Body weight was measured on a precision electronic scale with light clothing, without shoes, and height on a wall-mounted stadiometer. BMI was calculated, and diagnosis of overweight or obesity was made according to the 2023 American Academy of Pediatrics Clinical Practice Guidelines for the Evaluation and Treatment of Adolescents With Obesity, based on BMI percentiles of the CDC growth charts for age and gender (BMI ≥ 85th percentile defining overweight and BMI ≥ 95th percentile defining obesity) [22]. Waist Circumference (WC) was measured with a simple tape, midway between the lower ribs and the anterior superior iliac crests.

A morning blood sample was obtained from all participants, after an overnight fast. Fasting glucose, insulin, total cholesterol (TC), low-density lipoprotein cholesterol (LDL-C), high-density lipoprotein cholesterol (HDL-C), triglycerides (TGs), and uric acid were measured with standard methods (Cobas C 702, Cobas e801 analyzers). Glycated hemoglobin (HbA1c) was assessed by high-performance liquid chromatography [HPLC, AA-8121, Menarini Co., (Florence, Italy)]. After an overnight fast, an oral glucose tolerance test (OGTT) was performed in all participants after administration of 1.75 g/kg of anhydrous glucose (max 75 g) in aqueous solution. Blood samples were taken after 0′, 30′, 60′, 90′, and 120′ min of glucose administration to measure glucose and insulin levels. The Homeostasis Model Assessment for Insulin Resistance index (HOMA-IR) was calculated according to the formula: HOMA-IR = (Fasting glucose (FPG) mg/dL × Fasting insulin (FI) μU/mL)/405 [23].

### 2.3. The Adolescent Sleep Hygiene Scale (ASHS) Questionnaire

The ASHS questionnaire was proposed as an adolescent sleep assessment tool by LeBourgois et al. in 2005 [24], following a modification of the Children’s Sleep Hygiene Scale (CSHS) questionnaire first published in 2002 [25]. It consists of 28 questions that examine the quality of the adolescent’s sleep over the past month. The original version of the questionnaire examined 9 different conceptual domains: physiological (5 questions), cognitive (6 questions), emotional (3 questions), sleep environment (4 questions), daytime sleep (1 question), substances (2 questions), bedtime routine (1 question), sleep stability (4 questions), and bed or bedroom sharing (2 questions). Responses are given by adolescents on a Linkert scale with a score from 1 to 6 and with the following assignment: 1 = always, 2 = often, if not always, 3 = quite often, 4 = sometimes times, 5 = once in a while, and 6 = never. The total ASHS score is calculated as the average of the 9 domains, and a higher numerical score corresponds to better sleep hygiene. In 2013, the revised ASHS (ASHSr) was proposed with 24 questions divided into 6 domains: cognitive–emotional (6 questions), behavioral (3 questions), sleep stability (3 questions), sleep time (2 questions), physiological (5 questions), and sleep environment (5 questions) [26]. Cut-off limits (±2 SD, 25th and 75th percentile) were also set to a normal Gaussian distribution, corresponding to cut-off scores of 3.8 and 4.9, respectively. ASHSr values below 3.8 correspond to poor sleep hygiene [26]. The validation of the questionnaire in a Greek adolescent population was performed on the original questionnaire applied in the present study [27].

This study was approved by the Bioethics Committee of the School of Medicine of the Aristotle University of Thessaloniki (approval no. 159/2023-6/9.5.2023). Informed consent to participate in this study was given by all study participants and guardians.

### 2.4. Statistical Analysis

Statistical analysis was performed using Jamovi statistical software version 1.6.3. Continuous variables were tested for normal distribution after applying the Kolmogorov–Smirnov or Shapiro–Wilk test. Data are presented as mean ± standard deviation (SD) or medians with 95% confidential intervals. Differences between the studied groups of subjects were investigated using Student’s *t*-test or the non-parametric analog of the Mann–Whitney U test. Correlations between continuous variables were investigated using Spearman’s Rho correlation test. A linear regression model was applied to estimate the relationship between a dependent variable and one or more independent variables, with respect to issues of confounding. The level of statistical significance was set at *p* < 0.05.

## 3. Results

**Figure 1:**
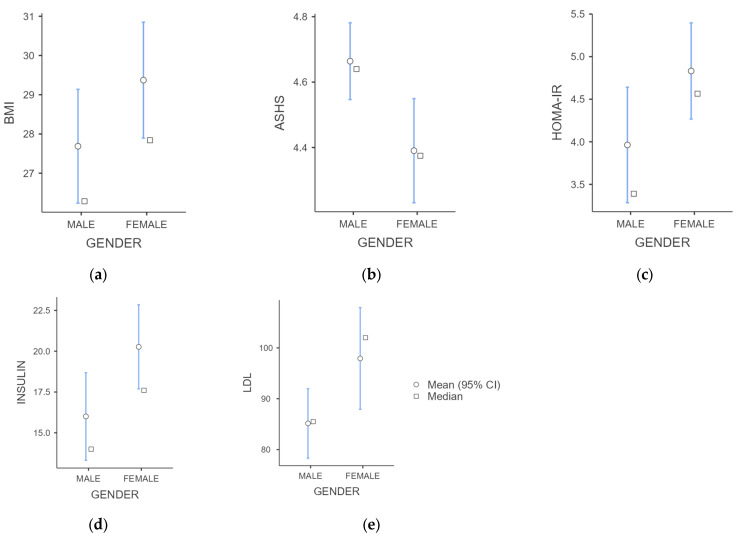
Mean, median, and 95% confidential intervals of different gender groups for the explored variables: BMI (**a**), ASHS score (**b**), HOMA-IR (**c**), fasting insulin levels (**d**), and LDL levels (**e**).

The study sample consisted of 100 participants, aged 13.80 ± 2.03 years. Among them, 48 out of 100 participants were males and 52 were females (Table 1). In the total sample, 56 (56%) participants were found with obesity and 44 (44%) with overweight. The average ΒΜΙ in the whole population was 28.26 ± 5.33 kg/m^2^, whereas females presented statistically higher BMI values compared to males (males: 27.69 ± 5.13, females: 29.37 ± 5.43, *p*-value: 0.049). Mean waist circumference did not present a statistically significant difference according to gender. The male gender presented statistically lower values of HOMA-IR (*p*-value: 0.008), fasting insulin (*p*-value: 0.003), and LDL (*p*-value: 0.009) compared to females (Figure 1).

The ASHS questionnaire scores revealed that there were gender differences. Specifically, it was found that the males had a statistically significant higher score than the females (*p*-value: 0.010).

The mean ASHS score exceeded the critical cut-off limit of 3.7 to define poor sleepers, both in the total sample and in the gender subgroups (Table 1).

The ASHS score in the nine domains of the questionnaire is analyzed in Table 2. The domains that collected the worst scores (in ascending order of scores) were sleep stability, the cognitive domain, and sleep routine. The results showed that the score of the last two domains was significantly worse than the others (*p* < 0.001), while no statistically significant difference was found between the two (*p* = 0.475). The domain of abuse was rated as the best mean in our sample. BMI appeared to be statistically significantly related to the following domains: phycological (r = −0.326, *p* < 0.001), cognitive (r = −0.245, *p* = 0.015), motional (r = −0.274, *p* = 0.006), substances (r = −0.350, *p*-value < 0.001), and daytime sleep (r = −0.216, *p*-value = 0.032).

**Figure 2:**
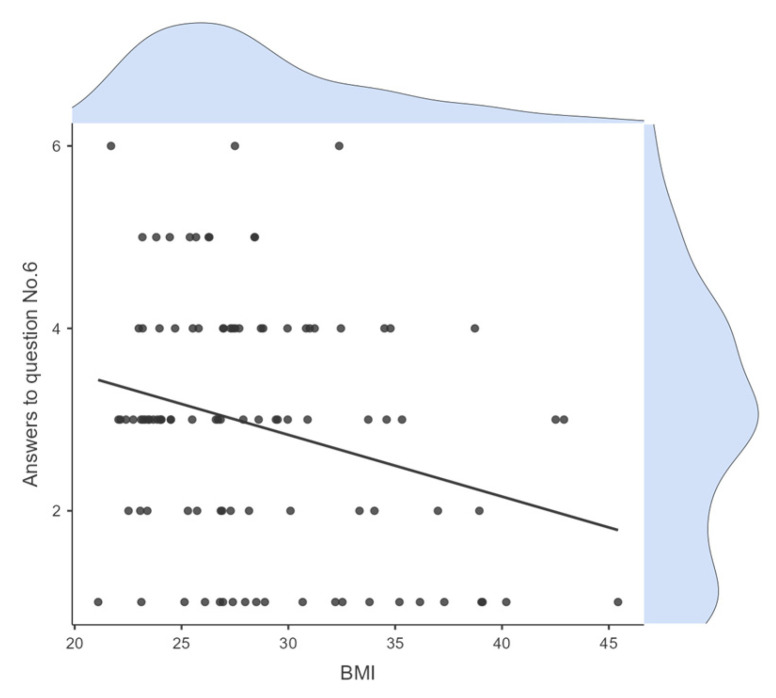
Correlation between answers on the Linkert scale to question 6 (“During 1 h before bedtime, I do things that make me feel very awake, for example, play video games, watch TV, talk on the phone”) and BMI.

Ιt is worth mentioning the adolescents’ answers to question 6 (“During 1 h before bedtime, I do things that make me feel very awake, for example, play video games, watch TV, talk on the phone”), which controls their screen use before bed. The score of these responses was negatively and statistically significantly related to BMI (r = −0.266, *p* = 0.008). Specifically, participants who answered that they follow this habit “often, if not always” (=2) or “always” (=1) have higher BMI values than those who answered “never” (=6) or “once in a while” (=5) (Figure 2). No correlation was found between these responses and other variables. In order to examine a possible association between body weight and social jet lag, it was also tested whether there is a correlation between BMI and the answers to question 26 “On the weekends, I stay asleep more than 1 h after my usual waking time”, but no correlation was found either with BMI (r = −0.001, *p* = 0.990) or other variables.

**Figure 3:**
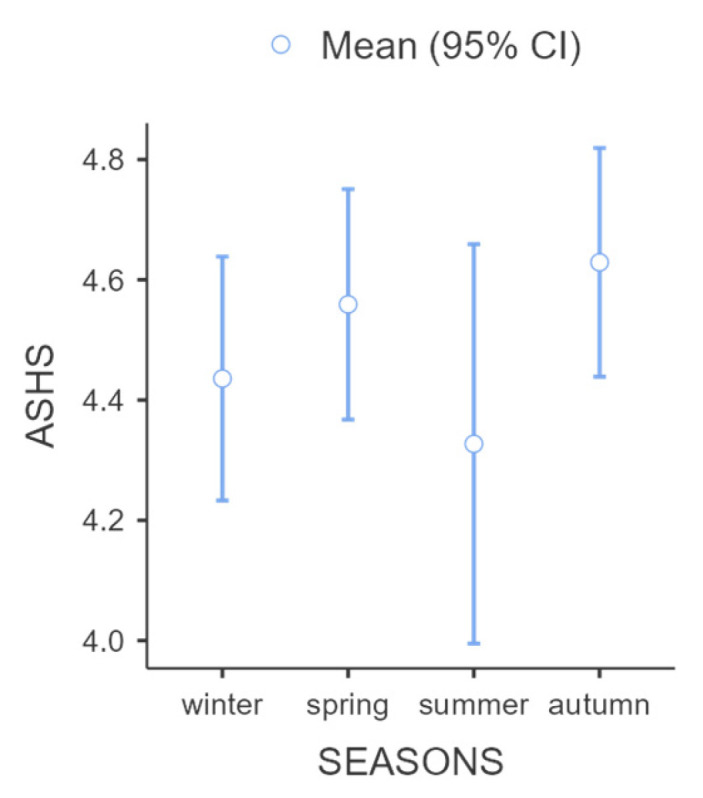
Sleep hygiene during the seasons of the year.

The responses of the participants were collected during the school term. Figure 2 describes the variation in the ASHS score by season [winter: 21 participants, winter ASHS score: 4.44 ± 0.46, spring: 35 participants, spring ASHS score: 4.56 ± 0.56, summer: 14 participants, summer ASHS score 4.33 ± 0.58, autumn: 30 participants, autumn ASHS score: 4.63 ± 0.51] (Figure 3). A paired *t*-test was conducted to explore the presence of a significant difference in sleep hygiene by season, and a statistically significant difference was found between spring and summer period ASHS scores (*p*-value = 0.024).

**Figure 4:**
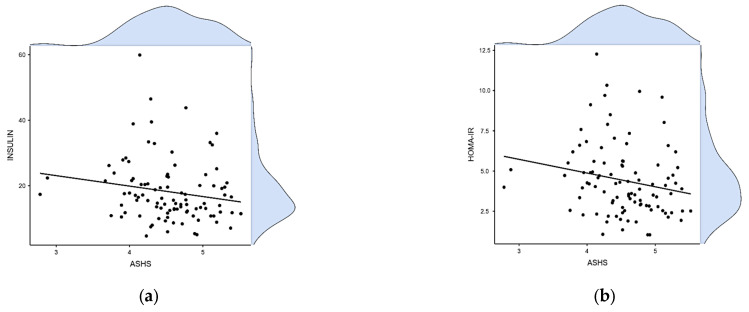
Correlation between ASHS score and insulin levels (**a**) and the HOMA-IR index (**b**).

**Figure 5:**
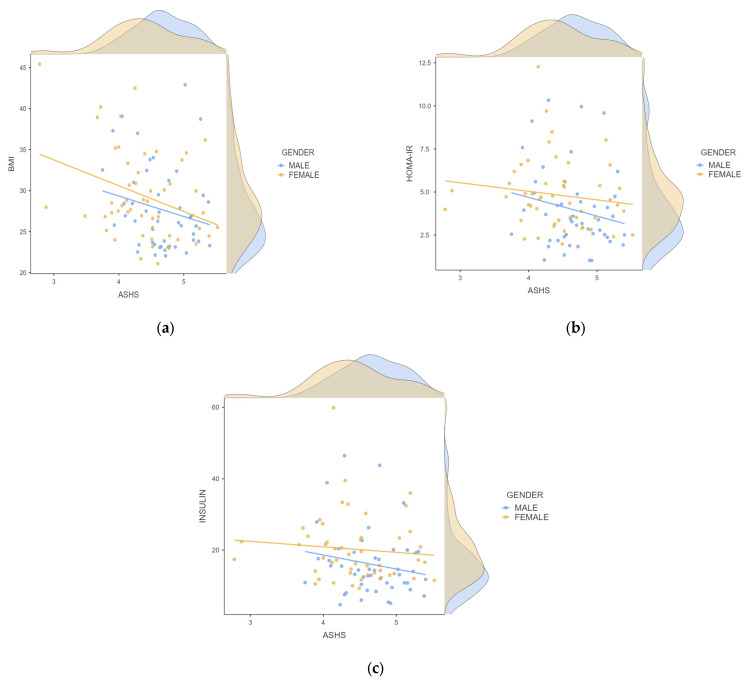
Correlation between the ASHS score and (**a**) BMI, (**b**) HOMA-IR, and (**c**) insulin levels by gender.

The correlation between the ASHS score and the various variables studied was investigated. A statistically significant negative correlation between adolescent sleep quality as expressed by the ASHS score and BMI, fasting insulin, and the HOMA-IR index was found (Table 3, Figure 4). A positive correlation was also noted between the ASHS score and TC values (Table 3). A linear regression model after adjustment for the variables gender and stage of puberty revealed that the ASHS score was strongly associated with the BMI variable (model coefficient: −2.168, SE: 1.05, *p*-value: 0.041) (Figure 5a). In contrast, the negative correlation between the ASHS score and insulin levels and the HOMA-IR index did not appear to be confirmed separately in the subpopulation of males (r = −0.153, *p*-value = 0.306 and r = −0.179, *p*-value = 0.230, respectively) or in the subpopulation of females (r = −0.174, *p*-value = 0.027 and r = −0.212, *p*-value = 0.139, respectively) (Figure 5b,c).

**Figure 6:**
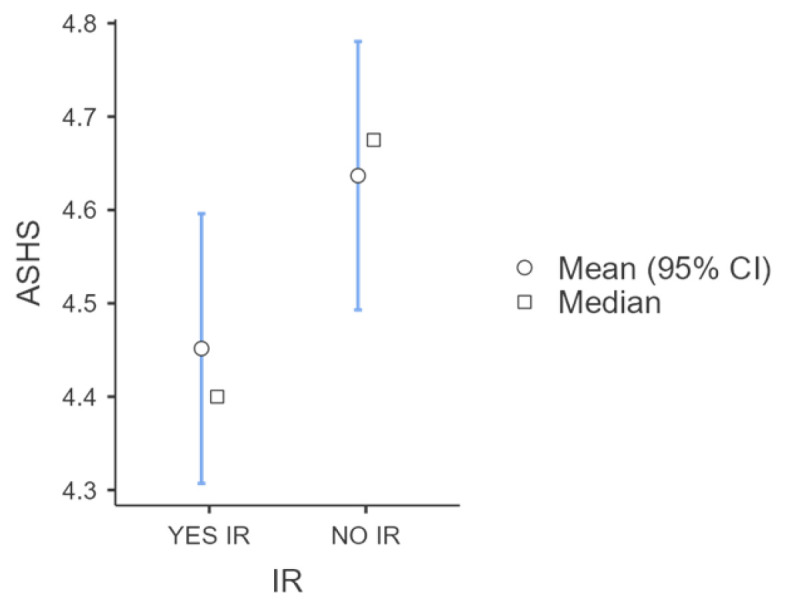
ASHS score among adolescents with and without insulin resistance, based on the HOMA index.

Participants were classified into two groups according to the presence or absence of insulin resistance (IR). Insulin resistance was defined as the HOMA-IR index exceeding either 2.5 for prepubertal participants (Tanner stage I) or 4 for pubertal participants (Tanner stages II–V). A total of 52.58% (n = 51/100, male = 20, female = 31) of the study population presented insulin resistance. Participants with IR presented a statistically higher ASHS score compared to the study population without IR (ASHS_IR_ score: 4.45 ± 0.53 vs. ASHS_non-IR_ score: 4.64 ± 0.50, *p*-value: 0.032) (Figure 6).

The effect of central obesity on the ASHS score was also explored. Study participants were grouped according to waist circumference percentile for age and gender (WC > 90th vs. WC < 90th). A total of 74.2% (n = 72/100) of the population presented with central obesity (WC > 90th percentile). No correlation was found between WC and the ASHS score in either the total sample or gender subgroups (ASHS WC > 90th score: 4.56 ± 0.49 vs. ASHS WC < 90th score: 4.51 ± 0.55, *p*-value: 0.566).

Menarche was also recorded in the subpopulation of girls. Menarche was evident in 30/41 females with a mean age of onset of 11.3 ± 1.22 years. A possible difference was explored for all variables studied in the present protocol, between menstruating girls (n = 30) and non-menstruating girls (n = 11). No statistically significant differences were recorded except for the ASHS score, where non-menstruating girls appeared to have better sleep hygiene compared to menstruating ones (*p*-value = 0.028).

**Figure 7:**
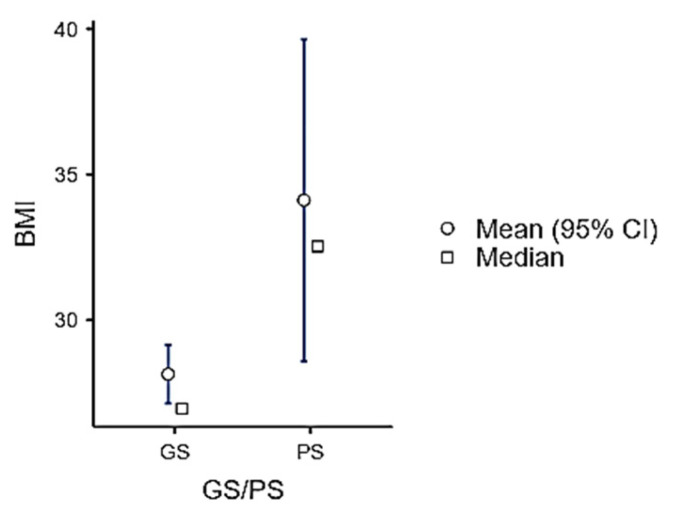
BMI variation between Good Sleepers and Poor Sleepers, defined by the ASHS score.

Participants were classified as Good Sleepers (GSs) or Poor Sleepers (PSs) based on their score on the ASHS questionnaire (cut-off score: 3.8). In the entire study population, 93% were classified as Good Sleepers (47 males, 46 females) and only 7% as Poor Sleepers. The Good Sleepers group presented a significantly lower BMI compared to Poor Sleepers (4.6 ± 0.44 vs. 3.44 ± 0.43, *p*-value: 0.026) (Figure 7).

## 4. Discussion

According to the results of the present study, the sleep quality of adolescents with obesity is significantly and inversely related to their BMI, fasting insulin levels, and the insulin resistance HOMA-IR index. Applying the ASHS tool, 93% of the sample are “Good Sleepers” while 7% of the sample is classified as “Poor Sleepers”. PSs had a statistically higher BMI compared to GSs. Among the nine domains assessed by the ASHS tool, the domains of sleep stability, cognitive level, and sleep routine scored the lowest among participants. The correlation between ASHS score and BMI remained significant after adjusting for the stage of puberty and gender of the participants (r = −0.026, *p* = 0.044). No significant association of sleep quality with abdominal obesity, fasting glucose levels, lipid profile, Hba1c, or uric acid was found.

During adolescence, body weight disturbances have been mainly associated with insufficient sleep duration and to a lesser extent with its quality [10,11,12,13,14,15,16,17,18,19,20,21,22,23,24,25,26]. In a clinical intervention study in obese adolescents, increasing sleep by one hour resulted in statistically significant reductions in body weight, WC, and insulin levels [28]. In a meta-analysis by Miller et al., short sleep duration was associated with a 1.3-fold higher risk of being overweight or obese (RR: 1.11–1.53), while every 1 h increased risk in sleep duration was associated with a 0.03 kg/m^2^ reduction in BMI [29]. Sleep quality was studied by Overberg et al. in adolescents with obesity [30]. Adolescents with unhealthy habits, such as sleeping late (late chronotype) or suffering from social jet lag (significant differences in bedtime between weekdays and weekends) had lower levels of melatonin secretion [30]. Low melatonin levels appear to be negatively associated with insulin resistance and obesity and have therefore been suggested as a risk factor for adolescent obesity [30].

In the present study, two sleep domains scored the lowest values among participants: sleep stability and cognition. Sleep stability reflected the difference in bedtime and wake time between weekdays and the weekend, while the cognitive domain corresponded exactly to pre-sleep thinking about the previous day’s events or the following day’s obligations, as stated. Although the latter domain initially appeared to have a significant association with BMI, when gender, age, and stage of puberty were taken into account as confounders, this relationship failed to be statistically significant.

Furthermore, in the present study, apart from the fact that sleep quality was significantly correlated with BMI in both genders, it was found that males had a higher total ASHS score compared to females but not statistically significant. However, it should be noted that in the present study, female participants presented a higher BMI compared to males. Similarly, in a study by Cespedes Feliciano et al. in a large sample of 804 adolescents, significant findings were demonstrated only for female adolescents: those classified as late or evening chronotypes by a rating scale appeared to have higher BMI and fat mass index (FMI) [31].

Moreover, it was estimated that for every one-hour increase in social jet lag, WC was 1.19 cm greater and FMI increased by 0.45 kg/m^2^ [31]. Regarding social jet lag, a study of 384 Mexican adolescents positively and independently correlated social jet lag with the HOMA-IR index. Similarly, this association was stronger for females [32]. In the present study, the association of ASHS score with insulin levels and the HOMA-IR index was confirmed as statistically significant. When the stage of puberty and gender were entered as cofactors in a linear regression test, the linear association was not confirmed. Therefore, there is an association, not a linear one, and this was also verified when patients were grouped based on HOMA-IR cut-offs, where insulin resistance and sleep quality were reported to be associated. In the review by Dutil et al., 13 of the 21 included studies supported the relationship of certain sleep characteristics (duration, architecture) with indicators of dysglycemia (hyperglycemia, insulin resistance), while one of the three studies found no association between glucose metabolism and sleep [15]. On the other hand, a study of 31 adolescents with obesity or overweight showed that social jet lag, delayed chronotype, and sleep duration of less than 6.5 h were associated with insulin resistance.

In addition, a multicenter cohort study in a European population of adolescents and children showed that sleep duration is directly and inversely related to WC and the occurrence of abdominal obesity, suggesting that there is no direct correlation between sleep duration and the HOMA-IR index, but only indirectly due to increased WC [33]. In our study, sleep quality was found to differ according to the WC of the participants when they were classified according to their WC percentile. In particular, it appeared that WC less than the 90th percentile achieved higher scores on the ASHS questionnaire and had better sleep quality. Assessing the relationship between sleep duration and insulin resistance, Javaheri’s group concluded that both short (5 h/night) and long sleep duration (10.5 h/night) were associated with an increase in the HOMA-R index by 25% compared to adolescents who slept about 7.5 h/day [34]. Thus, they proposed that the relationship between the two variables follows a U-shape morphology curve [34]. It should be emphasized that in their study, 20% of the adolescents were obese [34]. Similarly, the group of Koren et al., studying 62 adolescents with obesity, suggested that a U-shape relationship also describes the relationship between sleep duration and HbA1c or OGTT glucose values [35].

However, regarding increased sleep duration, there are conflicting data in the literature. A large study of children and adolescents of Chinese origin, which initially included 3200 participants (850 of them followed up after ten years), showed that only short sleep duration (less than 8 h/day) was associated with the occurrence of obesity or overweight and rejecting sleep duration greater than 10 h per day as a risk factor [36]. Similarly, another study in a Chinese population, by Duan et al., defined short sleep duration as <5 h/day and concluded that this duration increased the relative risk of developing metabolic syndrome [37]. In the present study, although long sleep duration was not assessed, participants were assessed as Good Sleepers and Poor Sleepers. The second category included 7/100 adolescents and appeared to have significantly worse sleep hygiene than GSs.

An important result that could also be considered is the fact that the mildly linear and negative relationship between the ASHS score and BMI is confirmed when comparing sleep between obese and overweight adolescents. Overweight adolescents appear to have better sleep hygiene than adolescents with obesity and this reflects the inverse relationship between weight and sleep.

Since the location of residence could be a confounding factor of sleep characteristics, data on the sleep patterns of the Greek population were retrieved from the literature. There are no relevant data available describing the same-origin (Greek) adolescent population, but there are data for Greek adults [38,39]. The prevalence of type 2 diabetes is associated with short sleep duration (<6 h/day), poor sleep quality, and feeling sleepy during the day [38]. Additionally, regarding the association of sleep with dyslipidemia, Greek adults demonstrate a significant association of the lipid profile with insomnia, reduced duration, and poor sleep quality [39]. In the present study, no correlation between lipids and adolescent sleep quality was found, and reliable international scientific data examining this specific position in the adolescent population are limited. An adolescent systematic review of seven eligible and highly heterogeneous studies attempted to assess sleep confounders, but only weak correlations were confirmed [40]. The authors concluded that if there is a correlation, it should be investigated in a weighted and targeted manner, as it would be a serious cardiovascular risk factor in adulthood [40]. More clinical intervention studies with very careful design regarding sample homogeneity and the absence of confounding factors are certainly needed.

The present study has some limitations that need to be addressed. The study population was derived from a single center, stratifying participants residing in Northern Greece, whereas the cross-sectional design of this study limits its ability to demonstrate causative relationships. However, the main strength of the present study is the fact that it focuses on the qualitative aspects of sleep, rather than just assessing the duration, while the application of the ASHS sleep quality score in the context of obesity has never been applied before among children and adolescents. Finally, this study represents the first investigation in a Greek pediatric population examining the relationship between sleep and body weight, glucose, and lipid metabolism. This is particularly relevant given the high prevalence of childhood obesity in Greece and the limited implementation of major interventions to address it.

## 5. Conclusions

Sleep quality, as reflected by the ASHS score, was negatively associated with BMI, fasting insulin levels, and HOMA-IR. A gender difference was noted in sleep quality in adolescence obesity and overweight since males achieved a higher total ASHS score compared to females. Adolescent “Poor Sleepers” had a significantly higher BMI than adolescent “Good Sleepers”. A widely accepted adolescent sleep assessment algorithm is necessary in order to draw firm conclusions about the association of sleep patterns and quality with glucose and lipid metabolism as well as energy balance.

## Figures and Tables

**Table 1:** Characteristics and ASHS scores in the total sample and gender subgroups.

	Male (n = 48)	Female (n = 52)	*p*-Value
Age (years)	13.60 ± 1.88	14.00 ± 2.15	0.306
BMI (kg/m^2^)	27.69 ± 5.13	29.37 ± 5.43	0.049
WC (cm)	94.90 ± 12.26	92.86 ± 11.96	0.414
Glucose (mg/dL)	97.81 ± 13.33	94.60 ± 11.96	0.310
Insulin (μIU/mL)	16.00 ± 9.35	20.27 ± 9.30	0.003
HbA1c%	5.28 ± 0.27	5.25 ± 0.33	0.616
HOMA-IR	3.96 ± 2.37	4.83 ± 2.04	0.008
TC (mg/dL)	151.69 ± 29.90	148.76 ± 31.31	0.517
HDL (mg/dL)	50.68 ± 12.04	46.98 ± 10.95	0.095
LDL (mg/dL)	85.16 ± 24.04	97.92 ± 36.78	0.009
TG (mg/dL)	79.10 ± 38.11	83.29 ± 25.39	0.237
non-HDL (mg/dL)	101.00 ± 28.35	101.76 ± 28.97	0.785
Uric acid (mg/dL)	5.18 ± 1.19	5.08 ± 1.25	0.612
ASHS Total Score	4.66 ± 0.42	4.39 ± 0.59	0.010

Variables are presented as mean ± standard deviation. *p*-value for the Mann–Whitney U test for comparison between gender groups. BMI, body mass index; TC, total cholesterol; TGs, triglycerides, LDL, low-density lipoprotein cholesterol; HDL, high-density lipoprotein cholesterol; ASHS, Adolescent Sleep Hygiene Scale.

**Table 2:** Mean score on the 9 domains of the ASHS questionnaire.

	ASHS Score Total	Domain 1 Bed/Bedroom Sharing	Domain 2 Phycological	Domain 3 Cognitive	Domain 4 Emotional	Domain 5 Sleep Environment	Domain 6 Substances	Domain 7 Sleep Routine	Domain 8 Daytime Sleep	Domain 9 Sleep Stability
Min	2.78	1.00	3.00	1.00	1.00	2.75	5.50	4.00	1.00	1.00
Max	5.52	6.00	5.80	5.17	6.00	6.00	6.00	6.00	6.00	6.00
Median	4.52	6.00	4.80	3.67	4.33	5.25	6.00	5.00	5.00	3.50
Mean	4.52	5.05	4.84	3.57	4.41	4.95	5.52	4.20	4.66	3.49
SD	0.53	1.45	0.59	0.95	1.13	0.82	0.78	1.74	1.31	1.03

**Table 3:** Correlations between study variables and the ASHS score in the total sample.

	ASHS	BMI	WC	Glucose	Insulin	HbA1c%	HOMA-IR	TC	HDL	LDL	TG	Non-HDL
ASHS	-											
	-											
BMI	−0.306	-										
	0.002	-										
WC	−0.101	0.834	-									
	0.330	<0.001	-									
Glucose	0.003	0.006	0.031	-								
	0.977	0.950	0.765	-								
Insulin	−0.224	0.355	0.224	0.055	-							
	0.027	<0.001	0.032	0.589	-							
HbA1c%	−0.095	0.016	0.116	0.137	0.092	-						
	0.359	0.879	0.272	0.182	0.379	-						
HOMA-IR	−0.260	0.369	0.274	0.324	0.888	0.087	-					
	0.010	<0.001	0.008	0.001	<0.001	0.405	-					
TC	0.202	−0.089	0.021	−0.234	0.083	0.031	−0.003	-				
	0.045	0.379	0.837	0.020	0.422	0.768	0.975	-				
HDL	0.061	−0.347	−0.272	−0257	−0.261	−0.094	−0.338	0.322	-			
	0.547	<0.001	0.008	0.010	0.010	0.367	<0.001	0.001	-			
LDL	0.087	0.069	0.096	−0.147	0.209	0.062	0.160	0.865	−0.033	-		
	0.392	0.498	0.355	0.144	0.040	0.547	0.117	<0.001	0.745	-		
TG	0.042	0.261	0.292	−0.088	0.335	−0.009	0.279	0.593	−0.286	0.663	-	
	0.682	0.009	0.004	0.388	<0.001	0.932	0.006	<0.001	0.004	<0.001	-	
non-HDL	0.154	0.057	0.144	−0.138	0.192	0.070	0.136	0.919	−0.040	0.944	0.747	-
	0.128	0.578	0.167	0.172	0.061	0.498	0.187	<0.001	0.696	<0.001	<0.001	-
Uric acid	0.061	0.335	0.366	0.004	0.147	−0.077	0.024	−0.080	−0.106	−0.108	0.111	−0.081
	0.613	0.004	0.002	0.971	0.220	0.526	0.843	0.507	0.378	0.370	0.358	0.503

Data are presented as Spearman’s rho value in the first line and *p*-value in the bottom line of each variable. Statistically significant correlations are highlighted as green when the correlation is positive and as red when the correlation is negative.

## Data Availability

The original contributions presented in this study are included in this article, further inquiries can be directed to the corresponding author.
